# Proteomic Analyses Identify Therapeutic Targets in Hepatocellular Carcinoma

**DOI:** 10.3389/fonc.2022.814120

**Published:** 2022-03-30

**Authors:** Abdulkadir Elmas, Amaia Lujambio, Kuan-lin Huang

**Affiliations:** ^1^Department of Genetics and Genomic Sciences, Center for Transformative Disease Modeling, Tisch Cancer Institute, Icahn Institute for Data Science and Genomic Technology, Icahn School of Medicine at Mount Sinai, New York, NY, United States; ^2^Department of Oncological Sciences, Liver Cancer Program, Division of Liver Diseases, Department of Medicine, Tisch Cancer Institute, Icahn School of Medicine at Mount Sinai, New York, United States

**Keywords:** hepatocellular carcinoma, proteomics, targeted therapy, precision oncology, proteogenomics

## Abstract

Hepatocellular carcinoma (HCC) is the fourth cause of cancer-related mortality worldwide. While many targeted therapies have been developed, the majority of HCC tumors do not harbor clinically actionable mutations. Protein-level aberrations, especially those not evident at the genomic level, present therapeutic opportunities but have rarely been systematically characterized in HCC. In this study, we performed proteogenomic analyses of 260 primary tumors from two HBV-related HCC patient cohorts with global mass-spectrometry (MS) proteomics data. Combining tumor-normal and inter-tumor analyses, we identified overexpressed targets including PDGFRB, FGFR4, ERBB2/3, CDK6 kinases and MFAP5, HMCN1, and Hsp proteins in HCC, many of which showed low frequencies of genomic and/or transcriptomic aberrations. Protein expression of FGFR4 kinase and Hsp proteins were significantly associated with response to their corresponding inhibitors. Our results provide a catalog of protein targets in HCC and demonstrate the potential of proteomics approaches in advancing precision medicine in cancer types lacking druggable mutations.

## Introduction

Hepatocellular carcinoma (HCC) is the sixth most common cancer and the fourth cause of cancer-related mortality worldwide (1). The currently FDA-approved available therapies include the multikinase inhibitors sorafenib (2), regorafenib (3), lenvatinib (4), and cabozantinib (5); the VEGFR2 antagonist ramucirumab (6), the immune checkpoint inhibitors pembrolizumab (7) and nivolumab (8) [alone or in combination with ipilimumab (9)], and the combination of atezolizumab and bevacizumab (10). Unfortunately, the survival benefits conferred by these treatments are typically limited to a few months. One grand challenge for identifying personalized and effective treatment options in HCC is the limited number of druggable mutations found in an average HCC patient (1). A compelling and underexplored strategy to identify novel drug targets and implement precision medicine for HCC patient is the discovery of aberrant protein targets not readily detected by genomic analyses that could serve as effective and selective drug targets.

Recent advancements in mass spectrometry (MS) technology have enabled the rapid expansion of global proteomic datasets that quantify almost the entirety of expressed proteins in primary tumor cohorts (11–18). The resulting proteomes of primary tumor cohorts provide ample opportunities for investigating protein-level aberrations that may be of clinical utility as prognostic biomarkers or therapeutic targets, including PAK1/PTK2/RIPK2 in breast cancer (19) and Rb phosphoprotein in colorectal cancer (13). However, protein aberrations have historically remained less characterized than genomic aberrations and systematic analyses to identify such targets are urgently needed (20–23). Further, upon the computational prioritization of protein targets, validation of their therapeutic viability requires a wide array of functional models representing inter-tumor heterogeneity observed across human tumors (24).

Herein, we identify and validate protein expression-driven therapeutic targets in HCC by utilizing recently generated global MS proteomic data from two human cohorts. Multiple kinases and other proteins showed up-regulated tumor expression and/or overexpression in primary tumors, and many of these targets show little evidence of DNA or RNA level alterations. Several targets including FGFR4 kinase and Hsp proteins further showed expression-driven dependency where the HCC cell lines with high protein expression were vulnerable to their respective targeting inhibitors. Overall, these results suggest that proteomic-based approaches could identify precision targets in HCC and cancer cases lacking actionable mutations.

## Results

### HCC Proteomics Cohorts

**Figure 1:**
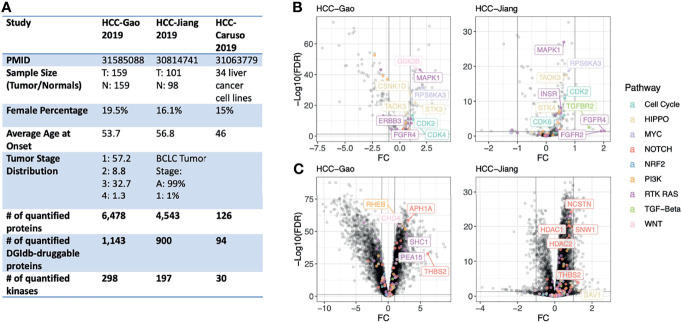
Study overview and differentially-expressed proteins in primary HCC tumors. **(A)** Overview of the proteogenomic datasets of human liver cancer cohorts and human liver cell lines analyzed in this study. **(B)** Volcano plots showing differentially-expressed kinase proteins between HCC tumor and normal liver samples in both the HCC-Gao and HCC-Jiang cohorts. The top differentially-expressed kinases from onco-signaling pathways are further labeled with text. **(C)** Volcano plots showing differentially-expressed non-kinase proteins between HCC tumor and normal liver samples in both the HCC-Gao and HCC-Jiang cohorts. The top differentially-expressed non-kinases from onco-signaling pathways are further labeled with text.

To test whether or not proteomics data could provide interesting drug targets for HCC we compiled genomic and global MS proteomic data from two cohorts of hepatitis B virus (HBV)-related hepatocellular carcinoma patients (**Figure 1A**). (1) The HCC-Gao cohort: the Gao et al., 2019 study of 159 cases with matched-normal samples (25), and (2) The HCC-Jiang cohort: the Jiang et al., 2019 study of 101 cases and 98 matched-normal samples (16). We applied standardized normalization procedure and quality-control criteria (Methods) and retained 6,452 quantified proteins in the HCC-Gao dataset and 4,500 proteins in the HCC-Jiang dataset. We also obtained a list of genes with corresponding drug compounds from the Drug-Gene Interaction database (DGIdb) (26); overlapping quantified proteins with this DGIdb druggable gene list, we identified 1,143 and 900 currently-druggable proteins in these HCC datasets, respectively. Given the higher coverage and larger sample size of the HCC-Gao cohort dataset, we present the HCC-Gao cohort’s findings as primary results and present the HCC-Jiang cohort’s findings second and confirmatory.

Oncogenic kinases are established therapeutic targets in multiple cancer types, and we further retained kinase proteins for subsequent analyses. Based on a previously curated list of 683 human kinase proteins (19, 27), the HCC-Gao and HCC-Jiang datasets included 298 and 197 well-quantified kinase proteins, respectively. Additionally, we annotated the proteins using ten oncogenic signaling pathways curated by TCGA PanCanAtlas, including the Cell Cycle, HIPPO signaling, MYC signaling, NOTCH signaling, oxidative stress response/NRF2, PI3K signaling, TGFβ signaling, receptor-tyrosine kinase (RTK)/RAS/MAP-Kinase signaling, TP53, and β-catenin/WNT signaling pathways (28).

### Differentially Expressed Proteins

For each cancer cohort, we performed a tumor-vs-normal paired analysis to identify differentially-expressed proteins (tumor-DEPs) by adjusting for potential confounding variables including age and gender using *limma* implementation in R (v3.42.2). DEP results from the HCC-Gao and HCC-Jiang cohorts showed concordance (**Figure S1**). In the HCC-Gao cohort, we identified 265 significant kinase DEPs (*limma* differential expression test based on the empirical Bayes moderation of the t-statistics, false discovery rate [FDR] < 0.05), of which 31 were annotated within an oncogenic signaling pathway. Among the kinase DEPs in the HCC-Gao cohort, 9 showed over 2-fold of up-regulation in tumors, namely, MAPK1 (log2-fold-change [FC] = 1.9, FDR = 7.3e-44), GSK3B (FC = 1.6, FDR = 1.5e-44), RPS6KA3 (FC = 2.1, FDR = 2.3e-32), STK3 (FC = 1.4, FDR = 1. 4e-22), CSNK1D (FC = 1, FDR = 2.9e-28), CDK2 (FC = 1.2, FDR = 2.5e-14), CDK4 (FC = 1.2, FDR = 2e-11), ERBB3 (FC = 1.1, FDR = 6e-12), and FGFR4 (FC = 1.1, FDR = 3.5e-9) (**Figure 1B**). Many of these kinases also showed significant up-regulation in tumors of the HCC-Jiang cohort, where for example FGFR4 kinase was also among the top-significant DEPs (FC = 2, FDR = 0.03) (**Figure 1B**).

Among the non-kinase proteins, we found 5,426 DEPs (FDR < 0.05) in HCC-Gao, of which 69 were annotated within an oncogenic signaling pathway. Among these, 18 showed over 2-fold of up-regulation, including THBS2 (FC = 6.1, FDR = 1.7e-33), APH1A (FC = 3.1, FDR = 3.6e-59), RHEB (FC = 2.7, FDR = 3e-52), SHC1 (FC = 2.8, FDR = 1.3e-48), and CHD4 (FC = 1.7, FDR = 6.8e-55) (**Figure 1C**). Notably, THBS2 protein was also significantly differentially-expressed (FC = 1.2, FDR = 1.3e-4) in the HCC-Jiang cohort (**Figure 1C**). HDAC1 and HDAC2 proteins showed up-regulation in tumors of both cohorts. The differential expression analyses discovered multiple proteins up-regulated in tumors compared to normal samples, and additional approaches are required to pinpoint therapeutic candidates.

### Protein Overexpression of Currently-Druggable Proteins

Many established protein targets in cancer (ex. HER2, EGFR, BRAF) are overexpressed in a fraction of tumor samples where their inhibition may show efficacy. To identify such overexpressed proteins in global MS proteomics data, we applied our recently-developed OverexPressed Protein and Transcript target Identifier (OPPTI) algorithm (29) (Methods), which is tailored to detect overexpressed proteins from global MS proteomic cohorts that may show varied quantitative distributions due to different technical platforms.

**Figure 2:**
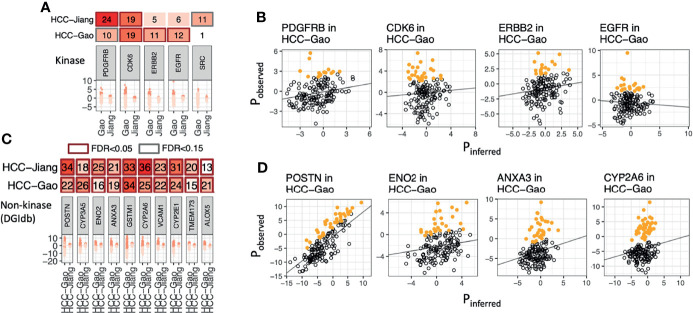
Overexpressed kinase and non-kinase proteins detected in human HCC tumors. **(A)** Protein kinases showing significant enrichment of overexpression as identified by OPPTI in either primary tumor cohort. **(B)** Sample-level kinase overexpression in HCC-Gao cohort of the markers shown in panel A, as identified by OPPTI through the deviation of observed protein expressions (y-axis) from the background inference (x-axis) and a cutoff value (not shown). **(C)** Ten non-kinase proteins showing the most significant enrichment of overexpression as identified by OPPTI in either primary tumor cohort. **(D)** Sample-level overexpression plots of the markers shown in panel C, as identified by OPPTI.

Applying OPPTI to the HCC-Gao cohort, we identified 46 kinases that showed significant enrichment of marker overexpression (OPPTI permutation test for overexpressed markers, FDR < 0.05), including CDK6 (Protein overexpression rate [PRO] = 18.9%, FDR = 1.6e-07), EGFR (PRO = 11.9%, FDR = 0.006), and ERBB2 (PRO = 11.3%, FDR = 0.013) (**Figure 2A**). In the HCC-Jiang cohort, we identified 33 kinases that showed significant enrichment of protein overexpression (FDR < 0.05), including CDK6 (PRO = 19.2%, FDR = 5.4e-05) and PDGFRB (PRO = 24.4%, FDR = 9.5e-07) (**Figure 2A** and **Figure S2**). To ensure the robustness of the identified targets, we calculated the concordance of overexpression frequency observed in the HCC-Gao and HCC-Jiang cohorts. The kinase overexpression rates identified by OPPTI showed a high correlation between the two cohorts (Pearson correlation test, R = 0.44, p = 5e-10), where CDK6 and PDGFRB displayed the largest overexpression rates among the potential HCC drug targets (**Figure S3**). Despite the intrinsic and technical MS differences between the two HCC cohorts, the coherency provided cross-validating evidence for the identified targets. Among the non-kinase proteins, 1,329 markers were significantly overexpressed (OPPTI permutation test, FDR < 0.05) in the HCC-Gao cohort, and among them 359 were DGIdb druggable genes. In HCC-Jiang cohort, 641 markers were significantly overexpressed (FDR < 0.05), and among them 161 were DGIdb druggable genes. Overall, we identified 100 non-kinase DGIdb druggable proteins that were significantly overexpressed (FDR < 0.05) in both HCC cohorts, including POSTN, CYP3A5, ANXA3, ENO2, and VCAM1 (**Figure 2C** and **Figure S3**).

**Figure 3:**
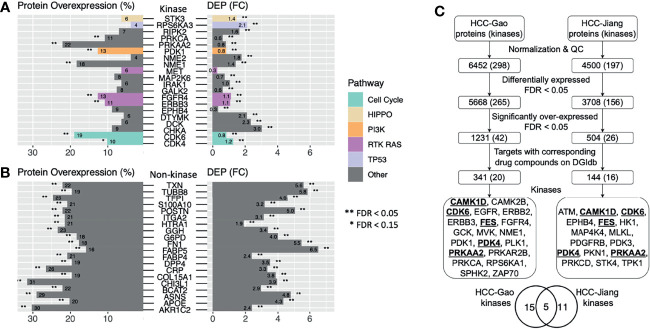
Candidate HCC protein targets showing protein overexpression, differential expression in tumor vs. normal tissues, and drug compounds as indicated by DGIdb. **(A)** Druggable kinases with corresponding drug compounds based on DGIdb that showed significantly higher tumor vs. normal expression and protein overexpression in the HCC-Gao cohort. **(B)** Druggable non-kinase proteins with corresponding drug compounds based on DGIdb that showed significantly higher tumor vs. normal expression and protein overexpression in the HCC-Gao cohort. **(C)** Flowchart showing the steps in the pipeline that generated the candidate HCC protein targets from two HCC cohorts.

While both DEP and overexpressed proteins present plausible methods to identify expression-based therapeutic targets, it remains unclear whether targets discovered by the two approaches overlap. We intersected the significant DEPs and the significant overexpressed markers to enhance confidence of identifying therapeutic targets (**Figure 3C**). In the HCC-Gao cohort, 187 kinases were quantified among the DGIdb druggable genes and 75 of them showed positive values in both differential expression and protein overexpression (**Figure 3A**). Among these, 3 kinases showed significant DE (limma differential expression test based on the empirical Bayes moderation of the t-statistics, FC ≥ 1, FDR < 0.05) and overexpression (OPPTI permutation test, FDR < 0.05), namely, NME1 (FC = 1.4, FDR = 5.0e-16; PRO = 18.2%, FDR = 1.6e-07), FGFR4 (FC = 1.1, FDR = 3.5e-09; PRO = 12.6%, FDR = 0.0026), ERBB3 (FC = 1.1, FDR = 6.0e-12; PRO = 10.7%, FDR = 0.027), as the RAS pathway (with FGFR4 and ERBB3 kinases) showed the most significant dysregulation. Other notable kinases were CDK6 from Cell Cycle pathway (FC = 0.8, FDR = 2.1e-4; PRO = 18.9%, FDR = 1.6e-07), and PDK1 from PI3K pathway (FC = 0.8, FDR = 1.5e-05; PRO = 12.6%, FDR = 0.0026).

Among the non-kinase targets we found 951 DGIdb druggable proteins quantified in the HCC-Gao cohort tumors, and 336 of them showed positive values in both differential expression and protein overexpression (**Figure 3B**). Among these, 68 kinases showed significant DE (FC ≥ 1, FDR < 0.05) and overexpression (FDR < 0.05), including, TXN (FC = 5.6, FDR = 2.7e-32; PRO = 22%, FDR = < 1e-100), POSTN (FC = 5, FDR = 4.6e-17; PRO = 22%, FDR = < 1e-100), and F5 (FC = 1.9, FDR = 8.5e-26; PRO = 13.8%, FDR = 4.1e-4). In HCC-Jiang cohort we found 774 quantified DGIdb druggable proteins, and 366 of them showed positive values in both differential expression and protein overexpression (**Figure S4**). Notably, 6 proteins showed significant DE (FC ≥ 1, FDR < 0.05) and overexpression (FDR < 0.05), including, POSTN and F5 (FC = 1.2, FDR = 1.5e-08; PRO = 34.2%, FDR = < 1e-100; and FC = 1.1, FDR = 2.3e-14; PRO = 16.5%, FDR = 7.8e-4, respectively), which were also identified in the HCC-Gao cohort. Several of the kinases have corresponding inhibitor drugs in clinical trials, and it remains to be validated whether the inhibitions of other DEP- and OPPTI-identified targets could serve as treatment strategies.

### Comparison Between DNA, RNA, and Protein-Level Alterations

**Figure 4:**
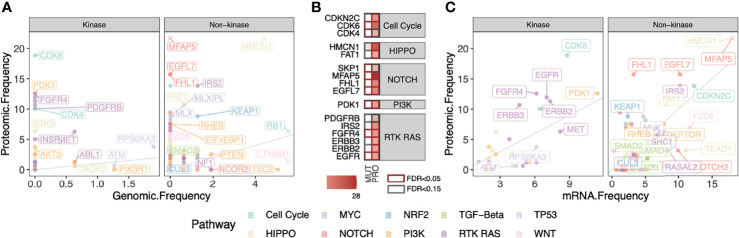
Comparison between fractions of cases carrying DNA, RNA, and Protein-level alterations in kinase and non-kinase targets in HCC. **(A)** Fractions of HCC cases carrying truncating or recurrent missense somatic mutations in the oncogenic signaling pathways compared to those showing protein overexpression in the HCC-Gao cohort. Top markers with high genomic and/or proteomic alterations are labeled. For better visualization of other data points, the outlying values of HMCN1 (original values: DNA = 4.4%, PRO = 22.6%), MFAP5 (DNA = 0%, PRO = 27.7%), RB1 (DNA = 5.7%, PRO = 6.3%), and CTNNB1 (original values: DNA = 18.4%, PRO = 1.3%), are truncated. **(B)** Proteins in panel A that show significant enrichment of protein overexpression (FDR < 0.15) tend to have low fractions of somatic mutations. **(C)** Fractions of HCC cases showing mRNA and protein overexpression frequencies of the genes in oncogenic signaling pathways in HCC-Gao cohort. The outlying values of HMCN1 (original values: RNA = 20%, PRO = 22.6%) and MFAP5 (RNA = 40%, PRO = 27.7%) are truncated.

Protein-level overexpression can arise from genomic alterations (i.e., copy-number amplification) but they may also arise post-transcriptionally and thus not readily observed at DNA or RNA levels. To examine these two competing hypotheses, we systematically compared the frequency of patients showing protein overexpression versus those carrying genomic mutations or transcriptomic aberrations. In the HCC-Gao cohort, we identified the fraction of cases having one or more recurrent missense or truncating mutations in the same genes. We then compared the fraction of HCC cases carrying these somatic mutations with those showing protein overexpression detected by OPPTI (**Figure 4A**). There were 127 genes with genomic alterations in the oncogenic signaling pathways with available protein quantification. HCC is known for the lack of actionable mutations, and as expected, no overexpressed kinases showed a genomic alteration rate greater than 5%. We thus investigated protein-level events that may arise independent of mutations. Five kinases from RAS pathway showed substantial protein up-regulation (PRO > 10%) with limited genomic alterations (likely driver), namely, ERBB2 (PRO = 11.3%, DNA = 0%), ERBB3 (PRO = 10.7%, DNA = 0%), PDGFRB (PRO = 10.1%, DNA = 0%), EGFR (PRO = 11.9%, DNA = 0%), and FGFR4 (PRO = 12.6%, DNA = 0%). (**Figures 4A, B**). Other proteins showing higher protein overexpression vs. driver genomic alteration rates include MFAP5 (PRO = 27.7%, DNA = 0%), HMCN1 (PRO = 22.6%, DNA = 4.4%), FHL1 (PRO = 15.7%, DNA = 0%), and EGFL7 (PRO = 15.7%, DNA = 0%). (**Figure 4B**).

We next compared protein overexpression to their respective mRNA overexpression by applying OPPTI with the same parameters to the RNA-Seq data available for the HCC-Gao cohort (Methods). We found two proteins with substantial rates of mRNA overexpression and protein overexpression, MFAP5 (mRNA overexpression rate [RNA] = 40%, PRO = 28%) and HMCN1 (RNA = 20%, PRO = 22.6%). We also found 4 proteins that showed significant protein up-regulation (PRO ≥ 10%) and higher (≥ 2-fold) protein overexpression rate than transcriptomic alteration rate, including CDK6 (RNA = 8.8%, PRO = 18.9%), FHL1 (RNA = 3.2%, PRO = 15.7%), FGFR4 (RNA = 6.1%, PRO = 12.6%), ERBB3 (RNA = 4.7%, PRO = 10.7%). (**Figure 4C**). Our results confirm the paucity of targets with genomic alteration in HCC and further demonstrate that a proteomic approach can uniquely identify a considerable fraction of overexpressed targets showing apparent aberrations at the protein level but not readily identified at the mRNA level.

### Validation of Therapeutic Efficacy Using Drug Screen Data

**Figure 5:**
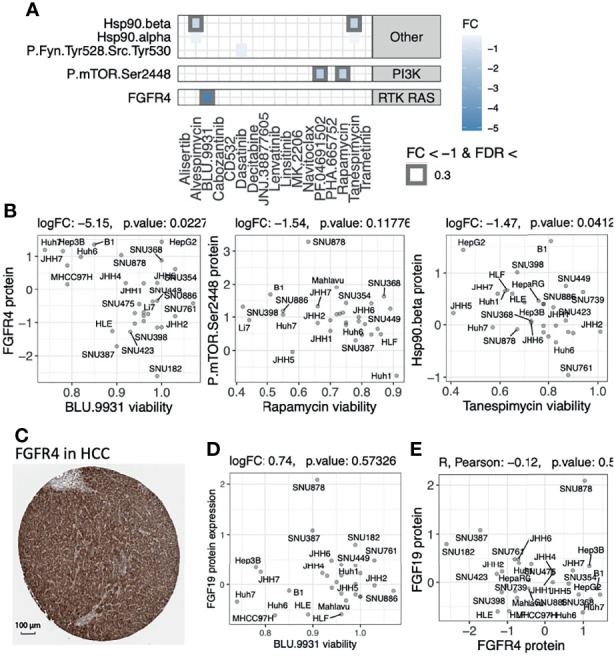
Evaluating the therapeutic efficacy of overexpressed protein targets found in primary tumors using drug screen data of human HCC cell lines. **(A)** Expression-driven dependency analyses highlight protein targets showing association between high protein expression and low cell viability upon treatment using drug compounds in a cohort of human HCC cell lines. **(B)** Scatter plots of the associations between cell viability and protein expressions of each compound’s respective target genes shown in panel **(A) (C)** IHC staining from the Human Pathology Atlas supporting the expression of the targeted kinase that showed significant expression-driven dependency. **(D)** The lack of correlation between BLU.9931 cell viability and FGF19 protein expression. **(E)** The lack of correlation between the protein expressions of FGFR4 and FGF19 genes in the HCC lines.

To validate the therapeutic potential of the protein targets that we identified in the primary tumor cohorts, we integrated the *in vitro* drug screen data of 31 anticancer agents on 34 human HCC cell lines available from Caruso et al. (30). For each drug, we analyzed the association between baseline protein expression levels (measured by Reverse Phase Protein Assay [RPPA]) and cell viability after treatment to identify expression-driven dependencies (Methods), where a negative association suggested HCC cells with high protein expression showed lower viability and were more vulnerable to the targeting drug. We first analyzed expression-driven dependencies of 40 genes encoding kinases that were known targets (antibodies) of the 31 screened compounds (30). We found several drug-protein associations (**Figure 5A**). FGFR4 expression was negatively associated with viability of cells treated with BLU.9931 compound (FC = -5.15, p-value [p] = 0.02, **Figure 5B**), validating the FGFR4 inhibitor’s efficacy in HCCs with up-regulated FGF19-FGFR4 signaling (31). FGFR4 protein expression was also orthogonally detected by immunohistochemistry (IHC) of HCC patient tumor samples in the human pathology atlas project (**Figure 5C**). MTOR expression (P.mTOR.Ser2448) was suggestively associated with the drug responses of Rapamycin (FC = -1.5, p = 0.12) and PF.04691502 (FC = -1.7, p = 0.19) (**Figure S5A**). Among the non-kinase targets, we found two associations between the expression of HSP90AB1 (Hsp90.beta) and the drug responses to Hsp90 protein inhibitors Tanespimycin (FC = -1.5, p = 0.04) and Alvespimycin (FC = -1.3, p = 0.07) (**Figure 5B** and **Figure S5B**). These results highlight FGFR4 and Hsp (HSP90AB4P) proteins as candidate therapeutic targets showing both up-regulation in HCC tumors and expression-driven dependencies.

FGFR4 is a receptor for the growth factor FGF19, whose up-regulation is thought to promote proliferation and tumorigenesis. The FGFR inhibitor BLU.9931 was previously shown to be effective against HCC xenograft tumors with amplified FGF19 (31). FGF19 protein expression (as evaluated by IHC) was further used to stratify patients for another selective FGFR4 inhibitor fisogatinib (BLU-554), where 17% (N=11/66) of the FGF19-positive patients responded compared to 0% (N=0/32) of the FGF19-negative patients (32). However, we did not observe a correlation between FGF19 upregulation and response to BLU.9931 in the human HCC cell lines (**Figure 5D**), which may be explained by the poor correlation between the FGFR4 and FGF19 protein expressions (**Figure 5E**). Analyzing data from the primary HCC tumor cohort, we also observed a lack of correlation between FGFR4 protein or mRNA expression and FGF19 gene expression levels (**Figure S6**). Phosphorylation data also showed a lack of correlation between FGFR4 (s573) phosphorylation and FGF19 gene expression levels but a strong correlation to FGFR4 protein expression (R = 0.57, p = 8.9E-13, **Figure S6**). These results imply that response to FGFR4 inhibitors and patient selection may be improved by using FGFR4 biomarkers in addition to FGF19 alone, and mechanistic intricacies in FGFR4/FGF19 signaling remain to be further determined.

## Discussion

We report a proteo-genomic evaluation of aberrant protein targets in 260 primary tumors from two HBV-related HCC cohorts (**Figure 1**). Tumor-normal and inter-tumor analyses of protein expression data highlighted multiple aberrantly-expressed protein targets in key signaling pathways, including PDGFRB, CDK6, ERBB2, and EGFR (**Figures 2**, **3**) whose protein overexpression in HCC tumors are also validated by IHC data from the Human Pathology Atlas (**Figure S7**). By integrating mutation, mRNA expression, and protein expression data, our proteogenomic analyses determined whether the overexpressed protein targets were concordant with genomic evidence or arose without genomic or transcriptomic alterations (**Figure 4**). Finally, the therapeutic viability of the identified targets was evaluated by analyzing drug screen data in human cell lines, implicating proteins whose up-regulation correlate with treatment response (**Figure 5**). These series of analyses have identified a list of prominent targets in HCC-Gao/-Jiang cohorts and the HCC-Caruso study (**Supplementary Tables 1, 2**).

Genome-based precision oncology in HCC poses a challenge where potentially targetable driver alterations are only identified in less than 30% of the patients (33). Proteomic analyses enabled us to identify new potentially targetable overexpressed proteins that may correspond to limited driver alterations, such as PDGFRB, ERBB2/3, EGFR and FGFR4 kinases upregulated in HCC tumors arising from no genomic driver alterations, as well as the non-kinase proteins such as MFAP5, HMCN1, EGFL7 and FHL1. Possible therapies for the overexpressed kinases include CDK4/CDK6 inhibitors, such as Palbociclib, which has been shown effective in human liver cancer cell lines and mouse models with intact tumor suppressor Retinoblastoma (Rb1) (34). ERBB2 could also be explored as a potential target in HCC, as evidence supports its involvement in liver tumorigenesis and intravenous injection of HER2-inhibitor Trastuzumab limited HCC growth *in vivo (* 35). Similarly, ERBB3 is overexpressed in hepatitis B-associated HCC, which are sensitive to ERBB3 inhibition (36). Erlotinib, an EGFR inhibitor, has been shown to be effective in patients treated with Lenvatinib as they upregulate EGFR, further supporting the role of EGFR as a biomarker (37).

By using human HCC cell lines that represent the heterogeneity observed in HCC patients, we evaluated the potential therapeutic efficacy of targets identified in primary tumors and showed protein expression of selected targets can predict treatment response. In particular, we found that the expression of FGFR4 kinase were significantly associated with drug response and may be a useful biomarker for FGFR4 inhibitors in addition to the currently-used FGF19 expression (31, 32). In addition, we observed a trend of improved recurrence-free survival in the HCC-Gao patients that did not overexpress FGFR4 protein compared to those overexpressing FGFR4 (p = 0.087; FGFR4-not-overexpressed median survival 23.2 months; FGFR4-overexpressed median survival 9.5 months) (**Figure S8**), although the association did not reach statistical significance and require validation in future larger-scale cohorts. Given the partial success of FGFR4 inhibitors in HCC patients, additional FGFR4 inhibitors have been developed and are under evaluation (38). Furthermore, tumors with elevated Hsp protein expression and MTOR phosphorylation may be more vulnerable to Hsp90 inhibitors and mTOR inhibitors such as rapamycin; other studies have also suggested that MTOR phosphorylation may be a better biomarker for mTOR inhibitors than genetic alterations in PTEN or TSC1/TSC2 (39).

In this study, the profiled primary HCC tumors collected in human cohorts are all related to HBV infection. This might pose a limitation as our findings may represent the HBV-specific features underlying the HBV-related HCC. Expanding the generalizability of the targets identified herein requires further investigation using the HCC cases related to different primary causes. The proteomic analyses of patient cohorts herein rely on global MS data, which can be time- and resource-intensive to generate in a clinical setting. Once the relevant protein markers are identified in these discovery studies, development of targeted assays using antibody-based (ex. IHC) or targeted MS technologies (ex. selected reaction monitoring) would be required.

To conclude, by employing a multi-omics approach, we investigated protein-level aberrations showing limited DNA or RNA level alterations in two human HCC cohorts and identified potential therapeutic targets showing expression-driven dependency upon targeting inhibitory treatment in human HCC cell lines; FGFR4 kinase and Hsp proteins, lacking actionable mutations, may be targetable in a fraction of HCC as supported by the vulnerability exposed by their respective targeting inhibitors. We believe that integrating proteomics data represents an unprecedented opportunity for the discovery of effective drug targets that may not be readily observed by genomic analyses in HCC and other cancer types.

## Methods

### Data Sources, Download, and Standardized Normalization

The proteomic and genomic datasets of HBV-related Hepatocellular Carcinoma (HCC-Gao cohort) were downloaded from The National Cancer Institute’s Clinical Proteomic Tumor Analysis Consortium (CPTAC) (25). This cohort contained 159 tumor samples with matched controls, and 6,478 unique proteins were quantified (of which 298 were kinases). The transcriptomic dataset was downloaded from https://www.biosino.org/node/project/detail/OEP000321. Proteomic and transcriptomic datasets of the other HBV-related HCC cohort (HCC-Jiang cohort) were downloaded from related publication (16). There were 101 tumor samples in the HCC-Jiang cohort with 98 matched controls. This cohort contained 7,878 unique proteins (of which 369 were kinases). The RNA-seq data contained gene expression profiles of 35 pairs of tumor and control samples, quantified by tophat-cufflinks pipeline. 16,457 protein-coding genes were identified with FPKM > 1 in more than one sample (of which 634 were kinase-encoding). For HCC-Jiang and HCC-Gao transcriptomics data, we used the quantile normalization and log2 normalization on the FPKM-normalized RNA-seq counts and filtered out genes showing no expression in at least 20% of the samples.

We examined the data distribution of each cancer proteomic cohort and performed a standardized normalization procedure for each dataset. Each sample within a cancer cohort is normalized by its Median Absolute Deviation (MAD), so that every sample across the datasets are normalized to unit MAD. We also filtered out protein markers with high fractions (at least 20%) of missing values.

### Identification of Differentially-Expressed Proteins

For each cohort, we performed a paired (tumor against matched-normal) analysis to identify differentially-expressed proteins by using the *limma* R package (v3.42.2). We corrected our analyses for confounding variables arising from batch effects when available (TMT batch, sequencing center/operator/date) or from demographics (age, gender), and the resulting p-values were multi-testing corrected using the BH procedure for FDR. For the majority of markers, we did not observe any significant confounding effect between protein expressions and the clinical variables age and gender (**Supplementary Table 3**); the only suggestive association was observed between the HSP09AB4P protein expression showing negative correlation with patient age in HCC-Jiang cohort (**Figure S9**, p=0.038 before multiple-testing correction).

### Detection of Overexpressed Proteins/Genes

To identify overexpressed markers, we used the OPPTI method (29). OPPTI is based on comparing expression levels to an inferred expression level in each tumor sample computed by a weighted k-nearest neighbor (KNN) algorithm, where the nearest features are the abundance level of other co-expressed markers. OPPTI performs a permutation test in order to evaluate the statistical significance of a marker’s potential enrichment of overexpression events. For a given cancer cohort, the dysregulation scores are permuted within every sample between the proteins, and the null overexpressions are computed from this data. After iterating this process multiple times, the null overexpressions accumulated from all iterations are used to establish the permutation distribution.

### Somatic Mutations and Comparison With Proteomic Overexpression

We reasoned that protein-truncating or recurrent somatic mutations were more likely to be functional. We thus retained all truncations (i.e., frameshift/non-frameshift deletion/insertion/substitution, stop-gain, stop-loss) present in the HCC-Gao cohort. Given the smaller size of this cohort, we also considered all missense mutations that have at least three occurrences in the open-access mutation call set files from the MC3 project of TCGA PanCanAtlas that applied standardized variant-calling pipeline and quality control processes (40).

### Analyses of Drug Screening Data in HCC Cell Lines

We downloaded the *in vitro* drug screen data on human HCC cell lines from Caruso et al. (30). We calculated the association of drug response with protein expression by using *limma* implementation in R (v3.40.6). For each drug, we performed the linear regression between the cell viabilities upon drug treatment and the targeted protein’s expressions across the drug-treated cell lines and obtained the corresponding coefficient of the linear fit. The resulting p-values were multi-testing corrected using the BH procedure for FDR.

## Data Availability Statement

The original contributions presented in the study are included in the article/**Supplementary Material**. Further inquiries can be directed to the corresponding author. OPPTI is available on GitHub: https://github.com/Huang-lab/oppti. Analyses were conducted based on scripts written using the R programming language version 3.6.2.

## Author Contributions

AE and KH designed the analyses. AE conducted the bioinformatics analyses and KH supervised the study. AE, AL, and KH wrote and edited the manuscript. All authors contributed to the article and approved the submitted version.

## Funding

This work was supported by Damon Runyon-Rachleff Innovation Award (DR52-18) and NIH/NCI R37 Merit Award (R37CA230636) to AL, as well as NIGMS R35GM138113 to KH The Tisch Cancer Institute and related research facilities are supported by P30 CA196521.

## Conflict of Interest

AL has received grant support from Pfizer and Genentech for unrelated projects.

The remaining authors declare that the research was conducted in the absence of any commercial or financial relationships that could be construed as a potential conflict of interest.

## Publisher’s Note

All claims expressed in this article are solely those of the authors and do not necessarily represent those of their affiliated organizations, or those of the publisher, the editors and the reviewers. Any product that may be evaluated in this article, or claim that may be made by its manufacturer, is not guaranteed or endorsed by the publisher.
