# Supplementary figures and images for: Proteomic Analyses Identify Therapeutic Targets in Hepatocellular Carcinoma
Source: Front Oncol. 2022 Mar 30;12:814120. doi: 10.3389/fonc.2022.814120 (PMC9006883; doi:10.3389/fonc.2022.814120)

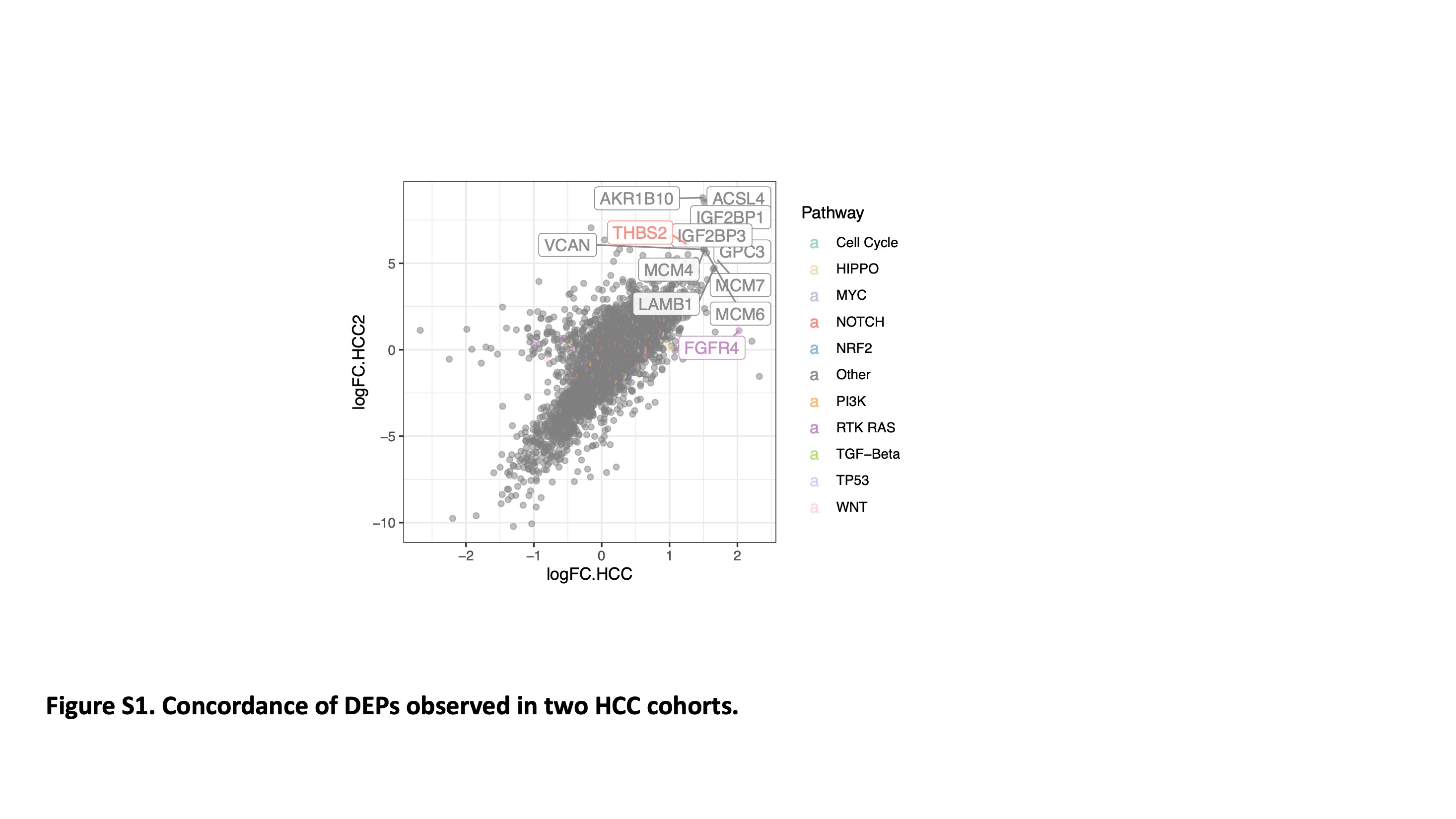

Supplement: Supplementary file 2 [file Image_1.jpeg]

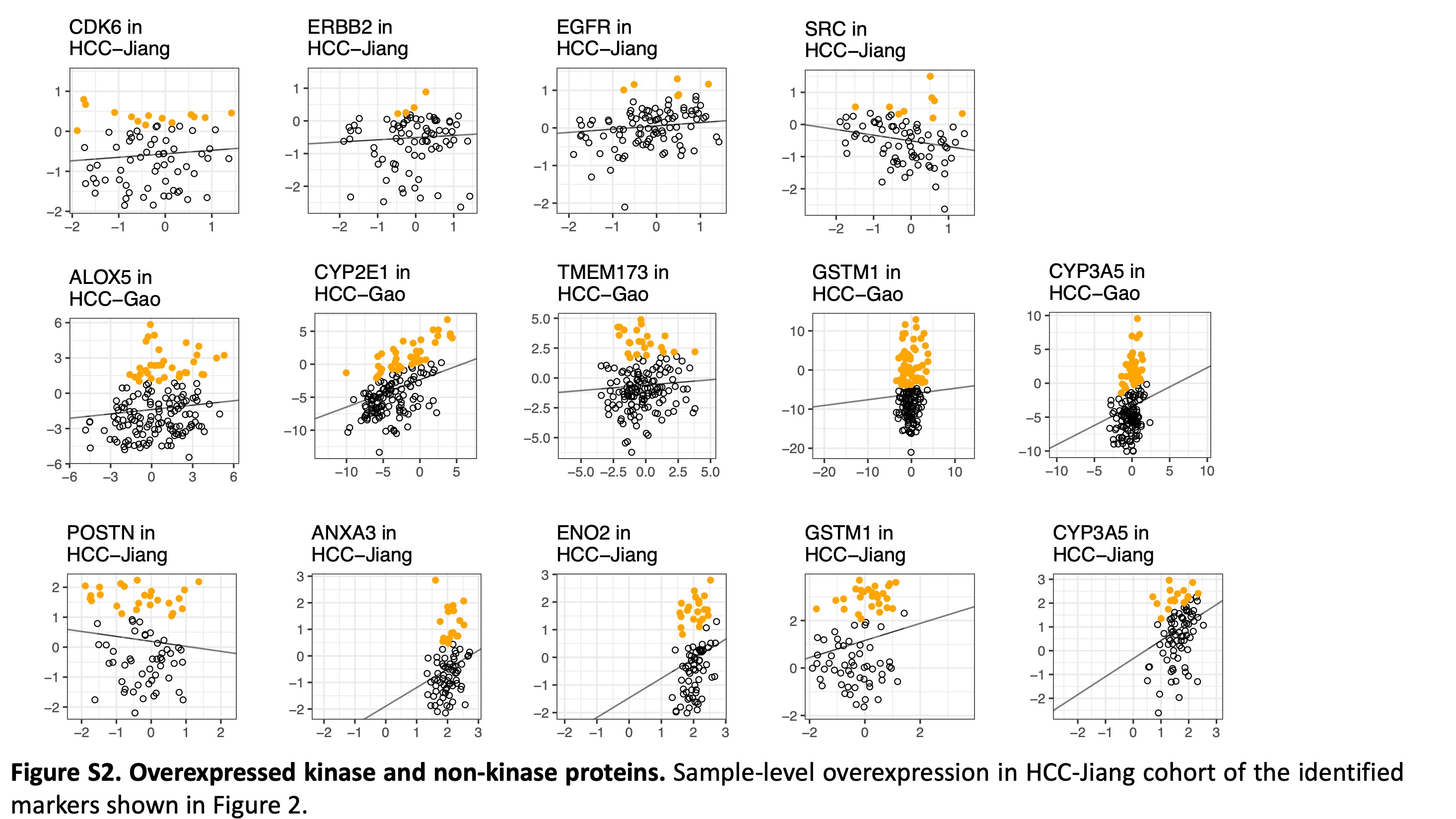

Supplement: Supplementary file 3 [file Image_2.jpeg]

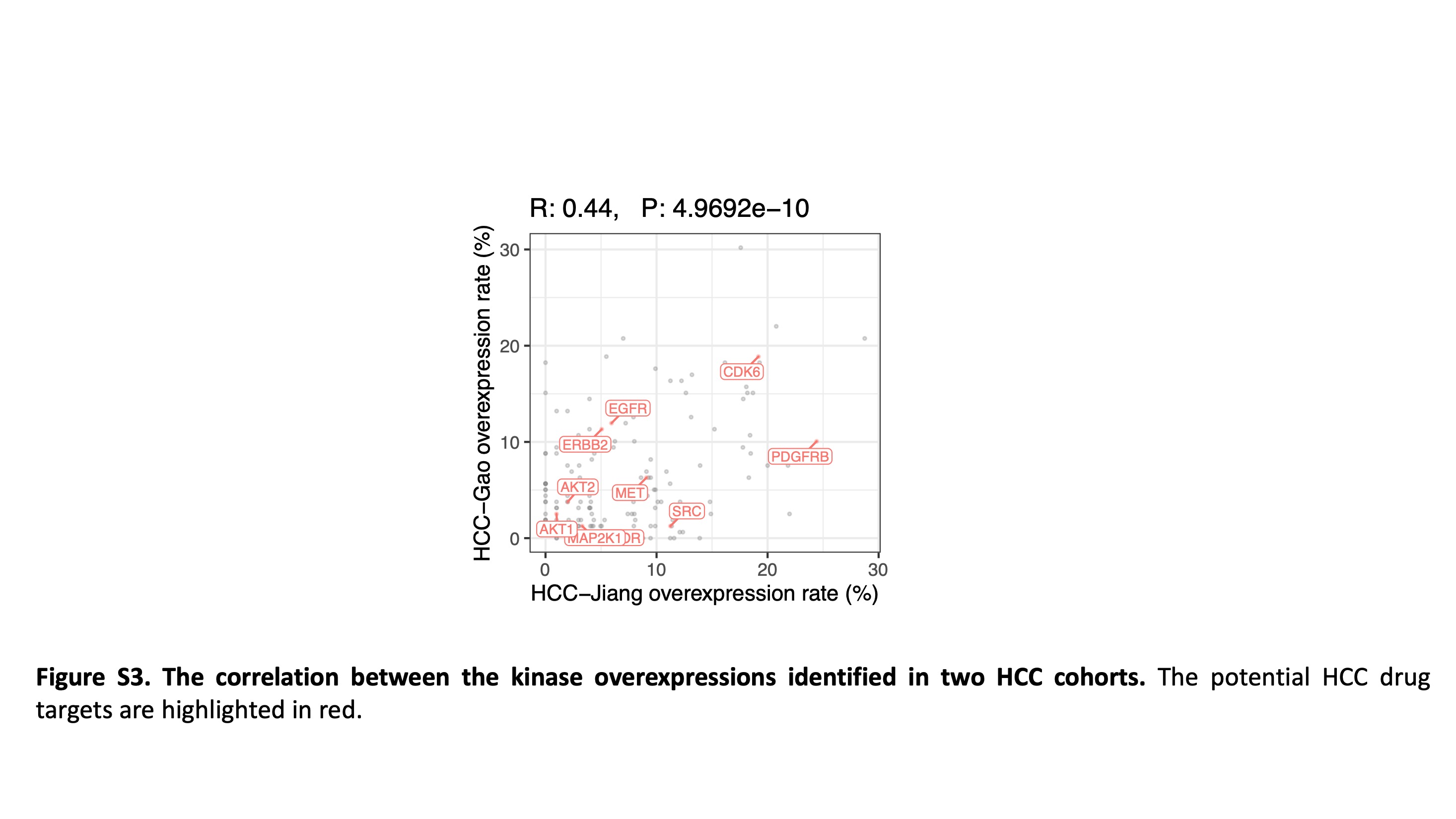

Supplement: Supplementary file 4 [file Image_3.jpeg]

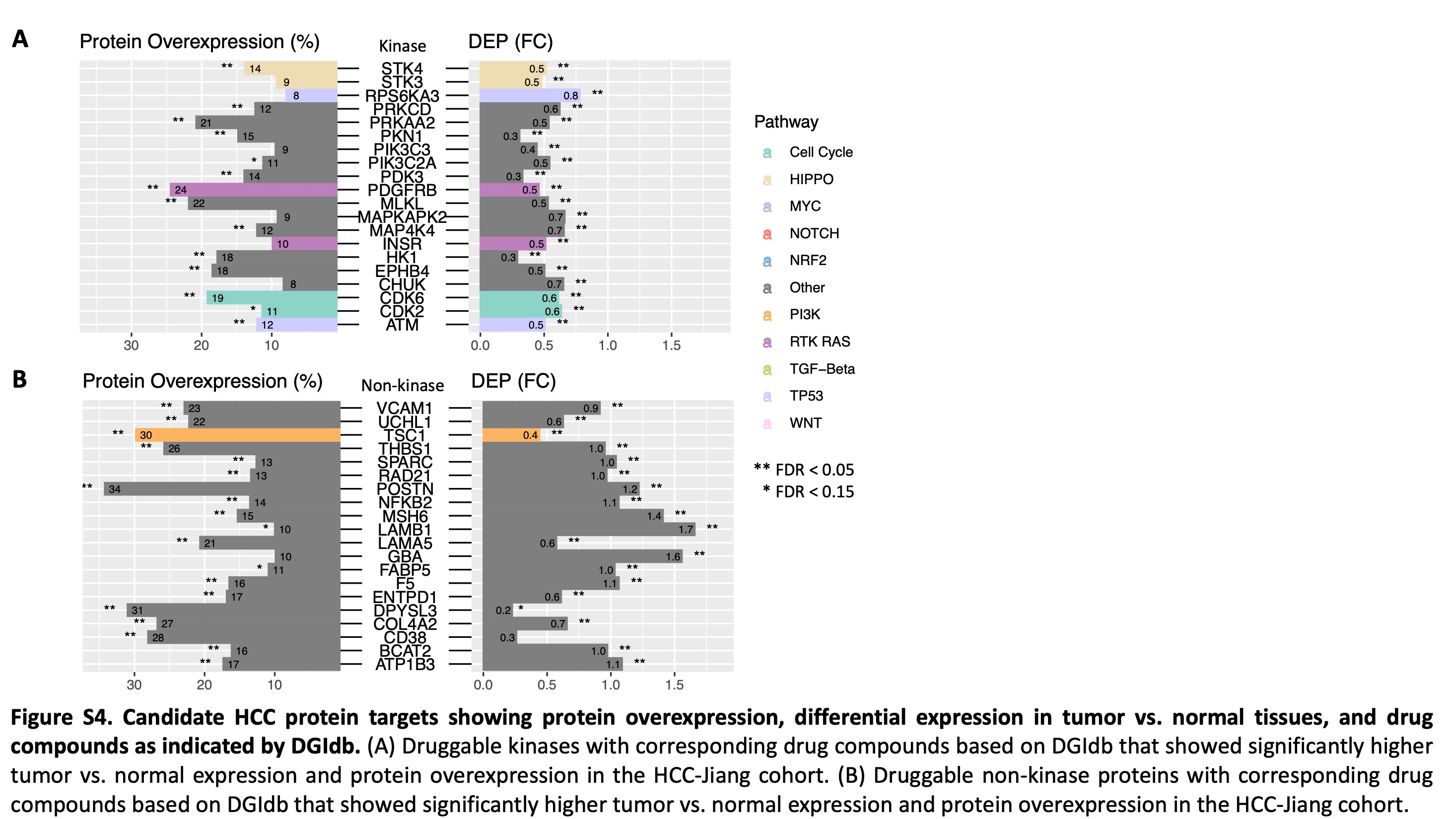

Supplement: Supplementary file 5 [file Image_4.jpeg]

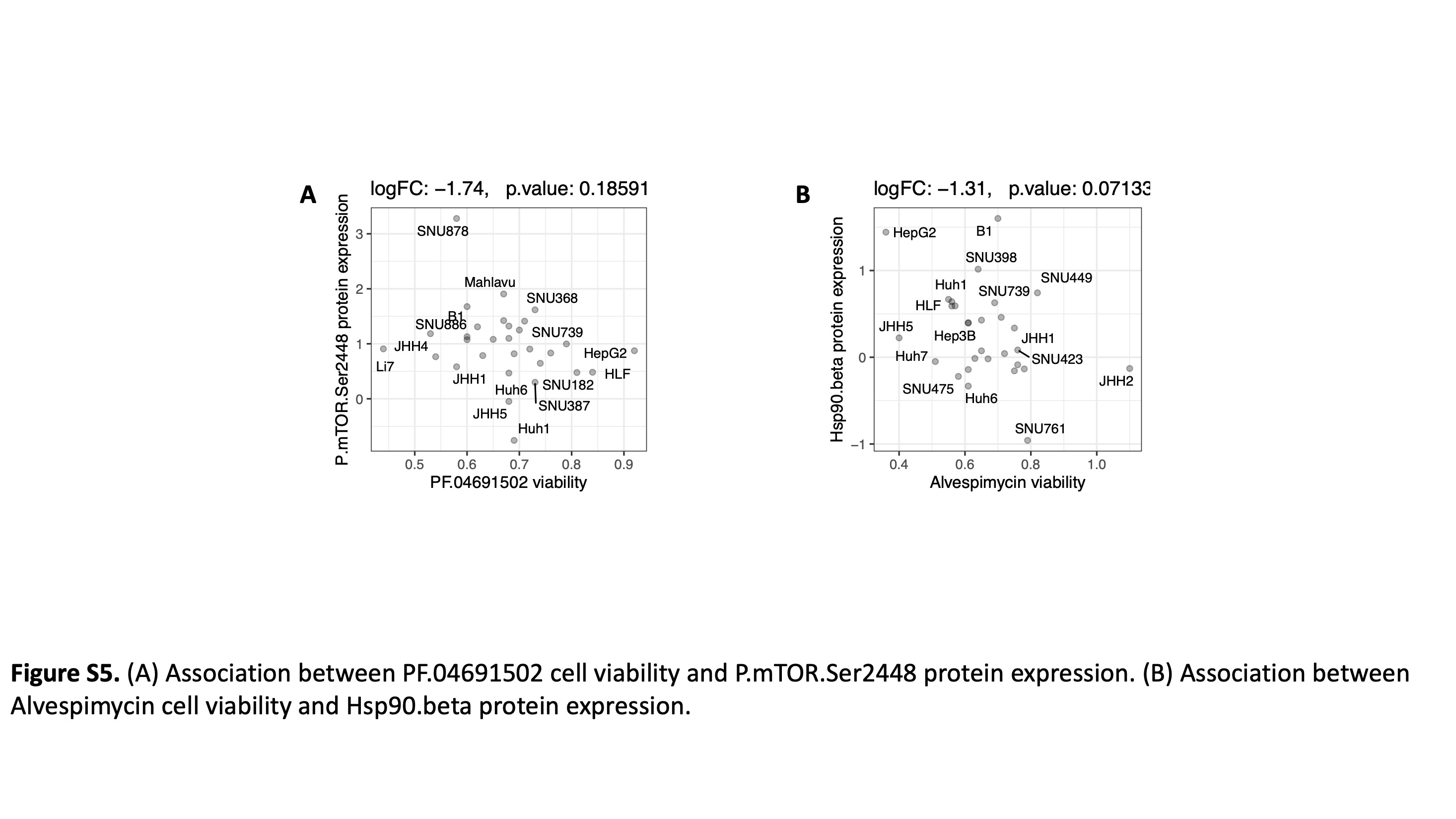

Supplement: Supplementary file 6 [file Image_5.jpeg]

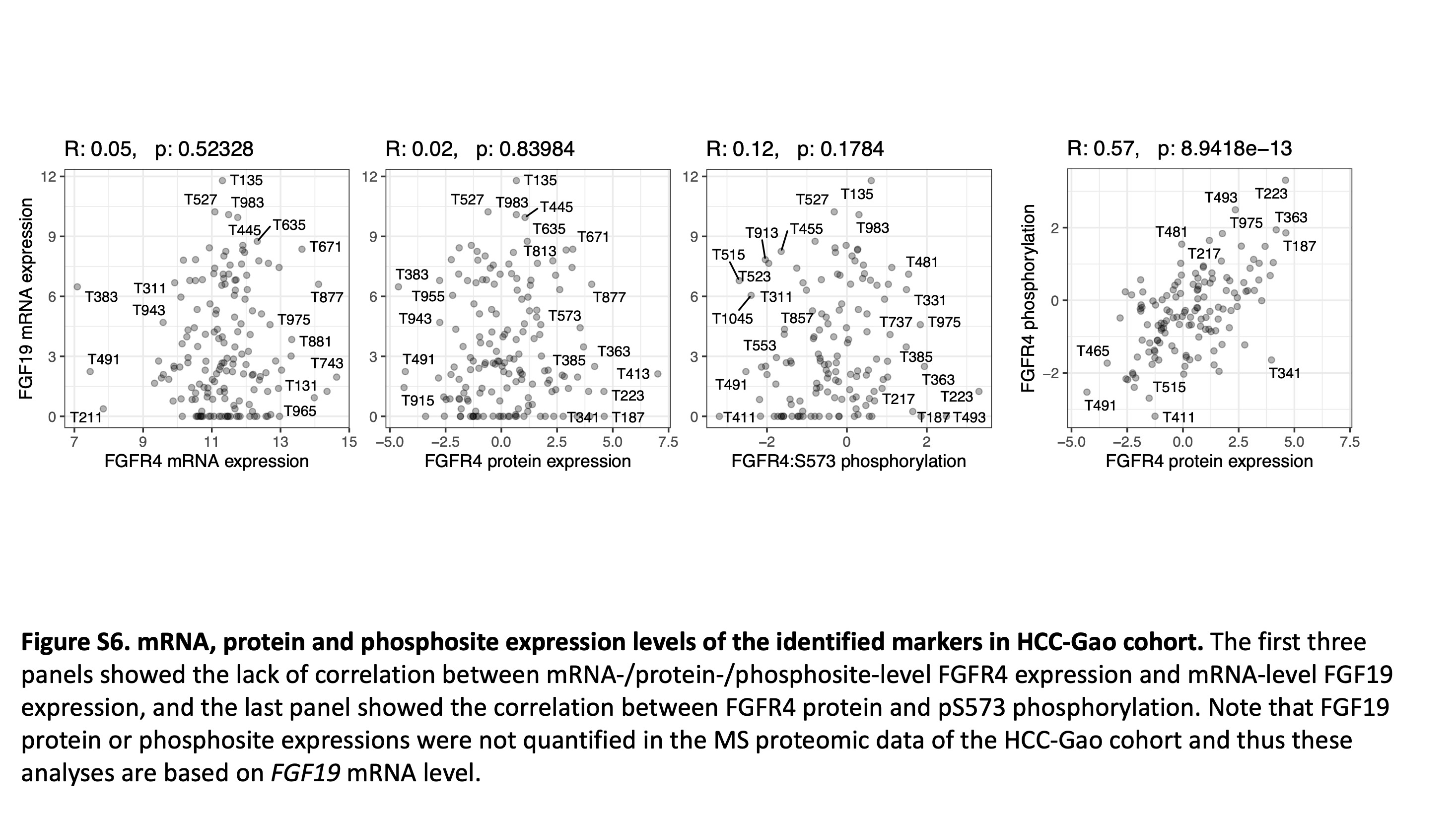

Supplement: Supplementary file 7 [file Image_6.jpeg]

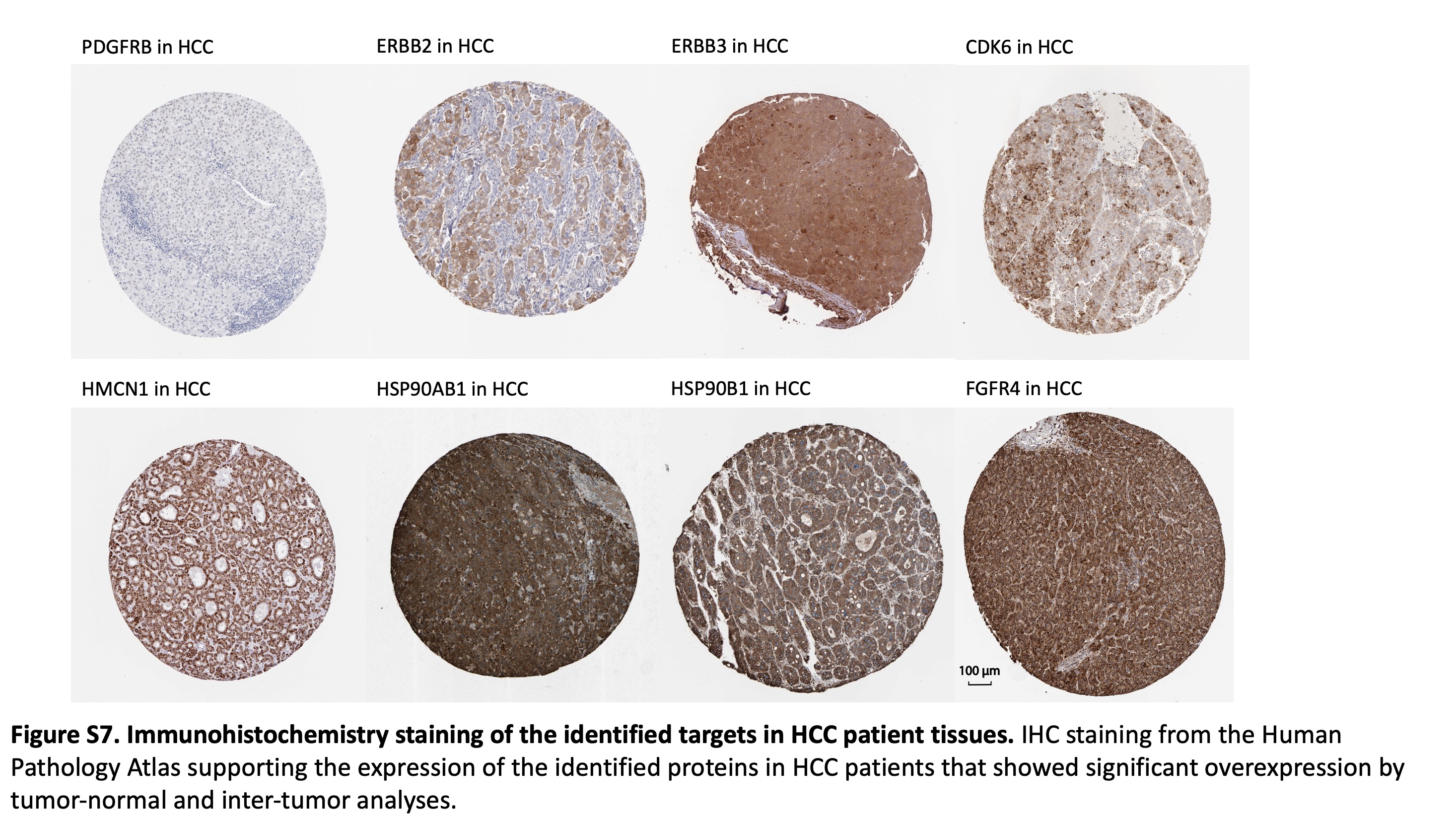

Supplement: Supplementary file 8 [file Image_7.jpeg]

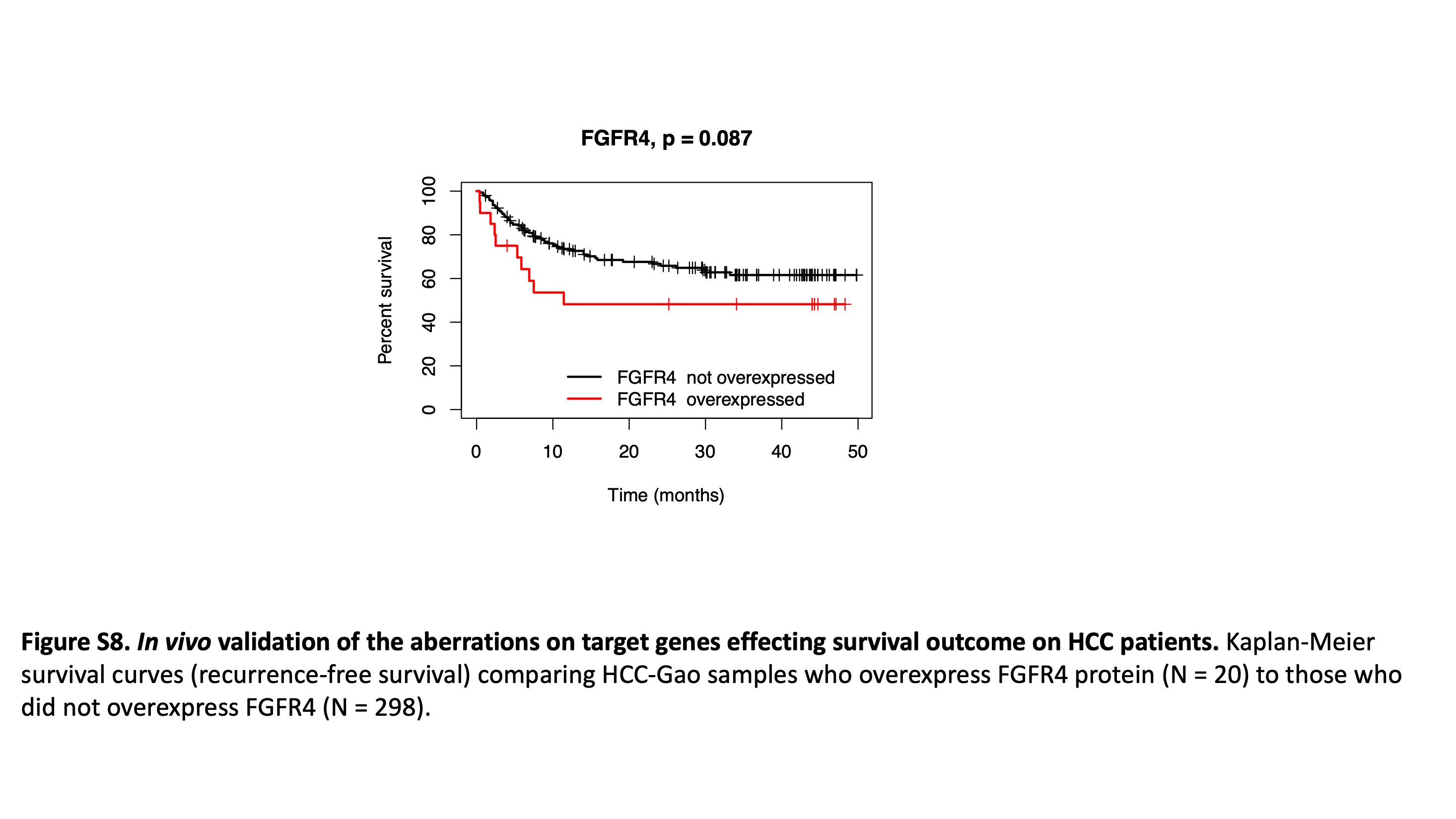

Supplement: Supplementary file 9 [file Image_8.jpeg]

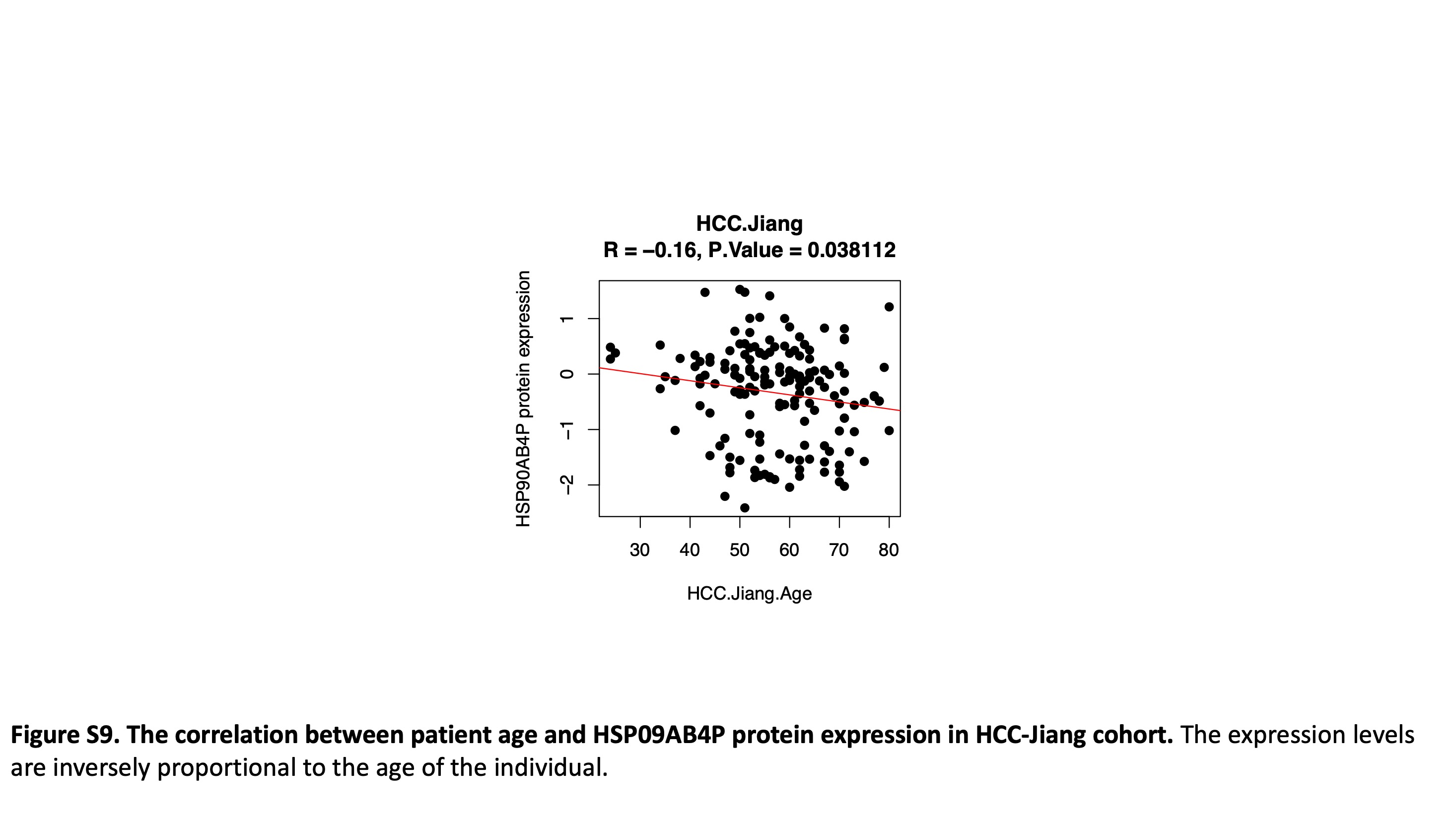

Supplement: Supplementary file 10 [file Image_9.jpeg]
